# Emotional prosody recognition is impaired in Alzheimer’s disease

**DOI:** 10.1186/s13195-022-00989-7

**Published:** 2022-04-05

**Authors:** Jana Amlerova, Jan Laczó, Zuzana Nedelska, Martina Laczó, Martin Vyhnálek, Bing Zhang, Kateřina Sheardova, Francesco Angelucci, Ross Andel, Jakub Hort

**Affiliations:** 1 Department of Neurology, Second Faculty of Medicine, Motol Epilepsy Center, Charles University, Motol University Hospital, Prague, Czech Republic; 2 Memory Clinic, Department of Neurology, Second Faculty of Medicine, Charles University, Motol University Hospital, Prague, Czech Republic; 3 International Clinical Research Center, St. Anne’s University Hospital Brno, Brno, Czech Republic; 4 Department of Radiology, Drum Tower Hospital of Nanjing University Medical School, Nanjing, China; 5 School of Aging Studies, University of South Florida, Tampa, FL USA

**Keywords:** Emotion recognition, Prosody, Alzheimer´s disease, Mild cognitive impairment, Temporal pole, Superior temporal sulcus

## Abstract

**Background:**

The ability to understand emotions is often disturbed in patients with cognitive impairments. Right temporal lobe structures play a crucial role in emotional processing, especially the amygdala, temporal pole (TP), superior temporal sulcus (STS), and anterior cingulate (AC). Those regions are affected in early stages of Alzheimer´s disease (AD). The aim of our study was to evaluate emotional prosody recognition (EPR) in participants with amnestic mild cognitive impairment (aMCI) due to AD, AD dementia patients, and cognitively healthy controls and to measure volumes or thickness of the brain structures involved in this process. In addition, we correlated EPR score to cognitive impairment as measured by MMSE. The receiver operating characteristic (ROC) analysis was used to assess the ability of EPR tests to differentiate the control group from the aMCI and dementia groups.

**Methods:**

Eighty-nine participants from the Czech Brain Aging Study: 43 aMCI due to AD, 36 AD dementia, and 23 controls, underwent Prosody Emotional Recognition Test. This experimental test included the playback of 25 sentences with neutral meaning each recorded with different emotional prosody (happiness, sadness, fear, disgust, anger). Volume of the amygdala and thickness of the TP, STS, and rostral and caudal parts of AC (RAC and CAC) were measured using FreeSurfer algorithm software. ANCOVA was used to evaluate EPR score differences. ROC analysis was used to assess the ability of EPR test to differentiate the control group from the aMCI and dementia groups. The Pearson’s correlation coefficients were calculated to explore relationships between EPR scores, structural brain measures, and MMSE.

**Results:**

EPR was lower in the dementia and aMCI groups compared with controls. EPR total score had high sensitivity in distinguishing between not only controls and patients, but also controls and aMCI, controls and dementia, and aMCI and dementia. EPR decreased with disease severity as it correlated with MMSE. There was a significant positive correlation of EPR and thickness of the right TP, STS, and bilateral RAC.

**Conclusions:**

EPR is impaired in AD dementia and aMCI due to AD. These data suggest that the broad range of AD symptoms may include specific deficits in the emotional sphere which further complicate the patient’s quality of life.

## Introduction

Emotion recognition (ER) plays a crucial role in interpersonal communication [1]. Emotional signals can be conveyed through different modalities including facial expressions, gestures, and voice or prosody (meaning melody, rhythm, rate, tone and loudness of speech) [1]. A deficit of ER can cause a series of problems ranging from disturbed interpersonal relationships to decreased quality of life [2].

ER includes a complex of behavior related to perception, motor mimicry, interoception, expression, and social judgment [3]. ER can be affected in a broad spectrum of neurological and psychiatric disorders [4], particularly in disorders where the brain structures responsible for those functions are typically impaired [5]. ER impairments have been demonstrated in frontotemporal dementia [6], Alzheimer’s disease (AD) and its prodromal phase of mild cognitive impairment (MCI) [7], Huntington’s disease [8], Parkinson’s disease [9], epilepsy [10], and traumatic brain injury [11].

According to the literature there is evidence that ER is connected with right temporal lobe structures [temporal pole (TP), superior temporal sulcus (STS), amygdala and anterior cingulate (AC)] [12–17]. Those structures are affected early during the course of AD [18, 19]; therefore, those patients may be at risk of ER deficit.

Assessments of ER have most often focused on facial ER using photographs of faces with different emotional expressions requiring participants to choose the proper emotion from a list. These studies usually test recognition of a core set of “basic emotions” [20], typically consisting of a group of negative emotions (e.g., anger, disgust, fear, sadness) and a single positive emotion (happiness).

In AD dementia and MCI, a deficit in facial recognition of emotions has been demonstrated but the data are often conflicting, with evidence of both impaired [21, 22] and preserved [23, 24] recognition. When considering specific emotions, the findings have been also inconsistent. While some studies report deficits in recognizing disgust, anger, sadness, fear, and happiness [25, 26], others report intact recognition for select emotions, such as disgust [27], anger [25, 28], and happiness [23, 24, 28].

Emotional prosody recognition (EPR) is a novel modality for experimental ER examination. EPR is frequently underutilized compared to other conventual ER tests, most likely due to its difficult testing protocol. Studies on EPR in AD have focused on sound properties of language, which can be demonstrated for example in the recognition of interrogative, notification, relative, and imperative sentences [29, 30].

In our study, we evaluated EPR using voice recordings in participants with amnestic MCI (aMCI) and dementia due to AD and compared these recordings with EPR in cognitively normal controls. Furthermore, we investigated the association between the EPR performance and either volume or thickness of selected brain regions, which are tightly connected with ER [13, 31].

We hypothesized that EPR

1. Is lower in aMCI and AD group and could be used as a clinical marker for early stages of dementia due to AD
2. Correlates with the volume/thickness of the right TP, amygdala, and STS and AC

To our knowledge, this is the first study evaluating EPR using real voice recordings in a large cohort of patients with aMCI and AD.

## Methods

### Participants

One hundred and two participants from the database of the Czech Brain Aging Study, a longitudinal, memory clinic-based study on aging and cognitive impairment [32], were investigated [33]. Of these, 43 participants were aMCI with high (30%) and intermediate (70%) biomarker probability of underlying AD pathology, 36 were diagnosed with dementia due to AD with high (25%) and intermediate (75%) biomarker probability of AD etiology, and 23 were cognitively normal participants. Biomarkers used included cerebrospinal fluid levels of amyloid beta, total tau, and phosphorylated tau proteins. In participants with aMCI, memory impairment was established when the participant scored more than 1.5 standard deviations below the mean of age and education-adjusted norms on any memory test, and activities of daily living were preserved to meet the Petersen et al. 2004 criteria [32, 34]. The aMCI group included both aMCI single-domain and aMCI multiple-domain phenotypes. All participants involved in this study signed written informed consent approved by the Motol University Hospital ethics committee.

### Exclusion criteria

Participants were excluded from the study if they reported a history of major neurological or psychiatric disorders, hearing difficulties, depression (≥ 6 points on the 15-item Geriatric Depression Scale) [35], or had significant vascular impairment on brain MRI (Fazekas scale more than 2) [36].

### Emotional Prosody Recognition Test

The Emotional Prosody Recognition Test was designed according to the methodology of Ariatti et al. published in 2008 [37]. The experimental battery was prepared in collaboration with four professional actors who produced 200 recordings in total using two Czech sentences with neutral meaning (“The table has four legs” or “Dogs that bark do not bite”). These sentences were spoken by two male and two female performers, native speakers, who were instructed to produce a specific emotional tone of voice (3-s duration) representing five emotions: sadness, fear, happiness, disgust, and anger.

From this large dataset the most appropriate emotions were chosen by 88 healthy volunteers recruited from the clinical staff of Motol University Hospital (mean age 31.6, M to F 1:1) to build the experimental battery. Volunteers invited to validate this test were relatively young because originally this test was prepared for examination of patients with epilepsy who are younger than patients with AD.

The final experimental test included 25 short recordings (spoken by one male and one female performer) each with a 3-s duration. Although the sentences held neutral semantic meaning, these recordings were presented with emotionally charged voices representing happiness, sadness, fear, disgust, and anger; thus, each emotion was represented 5 times. Recordings were presented to subjects on a computer using headphones. The 25 recordings were presented in the same order to each participant. Participants had to select the appropriate emotion from the list of emotions after each of the 25 recordings. There was no time limit to reduce stress during testing. When the participants hesitated, the examiner repeated the instruction to choose one answer from the list and waited until the participant made a choice. The test was scored as correct or incorrect after each recording and the maximum score was 25 points.

### MRI acquisition and analysis

Participants’ brain MR scans were performed on a 1.5T system (Siemens, Erlangen, Germany). A T1 weighted, 3-dimensional high resolution magnetization-prepared rapid acquisition with gradient echo (MPRAGE) was acquired with TR/TE/TI = 2000/3.08/1100 ms, flip angle 15, 192 continuous partitions, slice thickness 1.0 mm, and in-plane resolution 1 mm [38]. Participants’ scans were visually inspected to determine sufficient technical quality and to exclude participants with radiologic findings that could interfere with cognitive functioning (i.e., cortical infarctions, tumors, subdural hematomas, hydrocephalus or more extensive white matter hyperintensities equal to Fazekas scale above 2). To measure right- and left-sided amygdala volume and thickness of the temporal pole, superior temporal sulcus, and rostral and caudal parts of the AC (RAC and CAC), we used an automated algorithm from FreeSurfer, version 5.3. (http://surfer.nmr.mgh.harvard.edu), described in detail elsewhere [39, 40].

Amygdala volumes were normalized for the differences in head size by regressing the estimated total intracranial volume (eTIV) among participants, as previously described [41, 42]. Temporal pole, superior temporal sulcus, and RAC and CAC thickness were not eTIV adjusted.

### Statistical analysis

A one-way analysis of variance (ANOVA) with post hoc Sidak’s test was used to evaluate differences between the groups in continuous demographic variables. The *χ*^2^ test was used to evaluate differences in gender proportions. An analysis of covariance (ANCOVA) with post hoc Sidak’s test was used to evaluate differences between the groups in emotional prosody scores. The analysis was controlled for age (mean-centered) and years of education (mean-centered). The receiver operating characteristic (ROC) analysis was used to assess the ability of the Emotional Prosody Recognition Test to differentiate the control group from the aMCI and dementia groups. Sizes of the areas under the ROC curves (AUCs) and optimal sensitivity and specificity based on the Youden’s index were calculated. The Pearson’s correlation coefficients were calculated to explore the bivariate relationships between emotional prosody scores, structural brain measures, and Mini-Mental State Examination (MMSE) scores (a measure of disease severity). Holm-Bonferroni correction for multiple comparisons was used. Next, significant associations were tested using the hierarchical linear regression models adjusted for demographic characteristics, age (mean-centered), and years of education (mean-centered). A two-tailed *p* value < 0.05 was considered statistically significant. Analyses were performed using R statistical language environment [43] and IBM SPSS 25.0 software.

## Results

### Demographic characteristics

Group demographic and clinical characteristics are reported in Table 1. There was no difference in sex distribution among the groups. There was a significant group effect for age indicating that the control group was younger than the aMCI and dementia groups (both *p* < 0.001). There were no differences between the aMCI and dementia groups (*p* = 1.000).

**Table 1:** Demographic and clinical features of aMCI and AD patients and healthy controls. Data are expressed as mean ± standard deviation

Demographic and clinical features	MCI patients (***n*** = 43)	AD patients (***n*** = 36)	Normal participants (***n*** = 23)	***p***-value (group difference)
*Age*	74.47 **±** 6.09	74.47 **±** 6.54	67.04 **±** 6.67	0.00 *
*Gender*	26F/17M	25F/11M	17F/6M	0.836
*Years of education*	15.49 ± 2.83	13.47 **±** 3.56	16.65 **±** 2.37	0.00*
*MMSE*	26.60 **±** 2.57	22.75 **±** 2.55	29.52 **±** 0.73	0.00 *

*MMSE* Mini Mental State Examination, *M* male, *F* female

*Statistically significant

The dementia group had significantly fewer years of education compared to the control group (*p* < 0.001) and the aMCI group (*p* = 0.012). The difference in education between the control group and the aMCI group was not significant (*p* = 0.362). MMSE score was significantly lower in the aMCI and dementia groups compared to the control group (both *p* < 0.001). The dementia group had lower MMSE scores than the aMCI group (*p* < 0.001).

### Evaluation of emotional prosody recognition

**Fig. 1:**
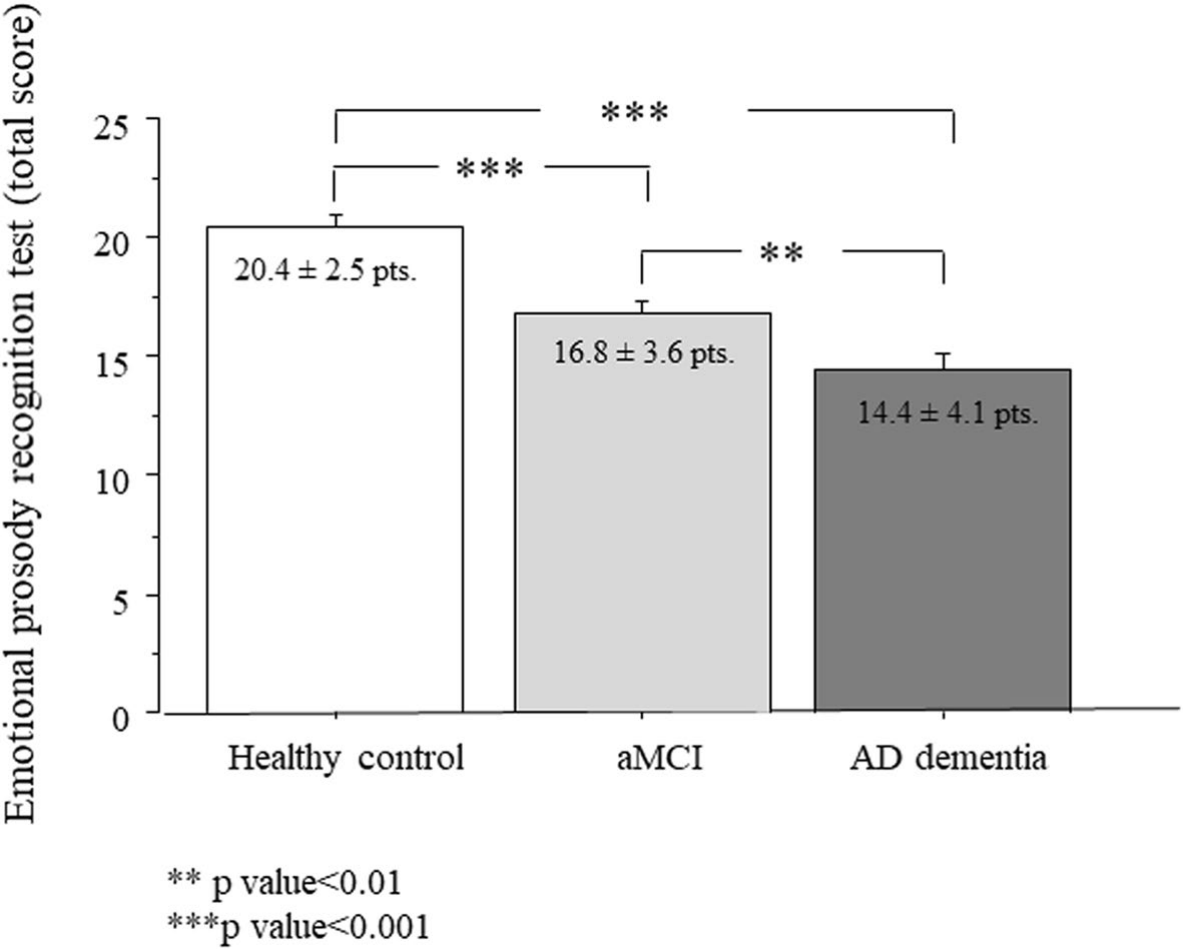
Prosody emotion recognition total score in aMCI and AD patients and healthy controls. Data are the mean ± SEM. Asterisk (*) indicates significant difference between the groups. **p < 0.001; ***p < 0.0001

The total score for EPR is shown in Fig. 1. There was a significant group effect in total EPR score (*p* = 0.002). The post hoc analysis controlling for age and education confirmed that EPR scores were lower in the dementia group compared to the control group (*p* = 0.002). The dementia group also had lower total EPR score compared to the aMCI group (*p* = 0.033) (Fig. 1).

In the ROC analyses, the total score for EPR differentiated the control group from the patients’ groups (pooled aMCI and dementia groups) with AUC values of 0.85 (*p* < 0.001), sensitivity of 87.0% and specificity of 72.2%, the control group from the aMCI group with AUC values of 0.80 (*p* < 0.001), sensitivity of 73.9% and specificity of 74.4%, the control group from the dementia group with AUC values of 0.91 (*p* < 0.001), sensitivity of 87.0% and specificity of 83.3%, and the aMCI group from the dementia group with AUC values of 0.67 (*p* = 0.009), sensitivity of 62.8% and specificity of 61.1%.

### Associations of emotional prosody recognition with MMSE and structural brain measures

EPR total score significantly correlated with disease severity according to MMSE score in the entire sample (*r* = 0.471, *p* < 0.001). The association remained significant in the regression analysis adjusted for age and education, where lower EPR total scores were associated with lower MMSE scores (*ß* = 0.35, *p* < .001, 95% CI [0.07, 0.27]).

EPR total score significantly correlated with left and right TP thickness (both *r* ≥ 0.35, *p* < .001), left and right STS thickness (both *r* ≥ 0.52, *p* < .001), and left and right RAC thickness (both r ≥ 0.35, *p* < .001). The correlation between ERP total score and left and right amygdala volumes (both *r* ≥ 0.23, *p* ≤ .026) did not survive the correction for multiple comparisons.

The associations with right-sided structural brain measures of TP and STS and bilateral structural brain measures of RAC remained significant in the regression analysis adjusted for age and education, where lower EPR total scores were associated with smaller right TP (*ß* = 0.33, *p* = .002, 95% CI [0.41, 1.71]), right STS (*ß* = 0.37, *p* < .001, 95% CI [1.32, 4.29]) and right (*ß* = 0.31, *p* < .001, 95% CI [1.85, 5.70]) and left (*ß* = 0.29, *p* = .001, 95% CI [1.39, 5.09]) RAC thickness. The associations between EPR total scores and left-sided structural brain measures of TP and STS were not significant in the regression analysis (all *ß* ≤ 0.14, *p* ≥ .199, 95% CI [− 0.39, 0.94] and [− 0.58, 2.76]).

## Discussion

This study investigated whether EPR is impaired in participants with aMCI due to AD and AD dementia as compared to cognitively normal participants. Secondly, we evaluated the correlations of EPR scores with volume of the amygdala and thickness of TP, STS, RAC, and CAC and cognitive deficit as measured by MMSE score. The ROC analysis was used to assess the ability of the EPR test to differentiate the control group from the aMCI and dementia groups. The results showed that EPR total scores were reduced in a group of aMCI and dementia as compared to cognitively normal controls. Also, EPR score was lower for the dementia group compared to aMCI. These results remain significant when age and education differences were controlled. ROC analysis showed that there was a high sensitivity to distinguish not only between controls and patients (dementia plus aMCI) but also between the controls and aMCI, controls and dementia, and aMCI and dementia groups.

In addition, the association between EPR and global cognitive functioning as measured by MMSE was also significant, with lower MMSE scores associated with a lower ability to recognize emotions from prosody (controlled for age and education differences). Several studies suggest that the disease severity may account for differences in ER between AD individuals and controls [44]. Therefore, it is important to consider the disease stage when analyzing ER in AD individuals.

Our findings indicate that EPR is impaired within the AD continuum ranging from dementia to prodromal stages of aMCI in a similar way to facial ER [45], and this aligns well with previous studies suggesting general ER impairment in AD [21, 22, 25].

In another study, it was shown that only facial ER is impaired in subjects affected by dementia of AD type, while EPR is unaffected [25]. This discrepancy with our data could be due to the low number of subjects included (*n* = 7). In addition, all subjects were diagnosed as MCI, thus excluding AD patients from their analysis. In another study [46] performed in a larger group of AD individuals with lower MMSE scores (19.9 ± 2.7), these participants showed a worse performance than controls in all ER tasks and particularly when identifying emotional prosody.

Thus, one possibility is that different results for emotional prosody in AD studies are due to recruitment of participants in different stage of the disease. This hypothesis is supported by our findings showing the positive correlation between MMSE and EPR total score. Current literature suggests that facial ER is impaired earlier than EPR during the course of the disease. Another explanation could be the possible heterogeneity of the sample used in those studies due to different methodology and AD diagnostic criteria used. Our study used biomarkers and extensive neuropsychology to define the AD and its prodromal stages, while a lot of studies depend only on MMSE staging.

The correlation with disease severity also suggests that assessment of EPR can be used as an additional tool to characterize the disease stage. There are studies using additional aspects of prosody or even complex music stimuli, and it has been recently proposed that speech sound analysis can be used to screen older adults for MCI or AD [47]. In another study, it was shown that subjects with dementia and aMCI also experience difficulty in recognizing the emotions conveyed by music [48]. The use of prosody score could be an important additional tool to stage AD combined with other classic methods like MRI, which is insufficient as a stand-alone tool [49].

In our study, correlation analyses showed positive correlations of EPR total score with thickness of TP, STS, and RAC. Controlled for between group differences, only right TP, STS thickness, and right and left RAC remain significant.

These brain structures, especially in right sided hemisphere, are involved in emotional processing in general. There are many studies supporting the role of the right hemisphere in emotional regulation [50]. For example, it has been shown that children with temporal lobe epilepsy, particularly those affected in the right lobe, have reduced EPR scores [51]. Similar data have also been found in adults [52]. It has long been thought that the superior temporal sulcus is connected with facial recognition; however, recent studies now show its importance also in perception of emotional prosody [53, 54]. By using real-time functional magnetic resonance imaging (rt-fMRI) techniques, it has been confirmed that the ACC is a central hub for cognitive and emotional networks [55] and its modulation has been suggested to elicit mood changes [56]. Moreover, analysis of the cerebral activity maps obtained by fMRI during EPR tests showed that these brain areas share a neural substrate for mentalizing and processing verbal and prosodic emotional cues [57]. These results are in line with current understanding of the right hemisphere to be involved in emotional processing in general [50, 52].

The correlations with volumetric data of distinct brain regions provide additional support for the use of EPR scores as a diagnostic tool. Specifically, we observed a positive correlation with right TP and STS thickness and bilateral RAC thickness, which are regions primarily involved in ER [58–60] and also affected early during the course of AD [16, 18, 19].

After the correction for multiple comparisons, the amygdala volume was not correlated to EPR score. Reduction in hippocampal and amygdala volume on structural MRI is considered to be an early marker of AD [61]. Although the amygdala is associated with emotional processing and generating emotional responses to presented faces [62], studies are not consistently in agreement on whether amygdala atrophy is present in AD versus controls [63, 64]. Furthermore, amygdala function in regulating and sustaining emotional processing is probably independent from the actual amygdala volume.

### Limitations

One possible limitation of our study is the significant difference in age and education between controls and individuals with aMCI and dementia. By adjusting our analyses for age and education we tried to address this issue. Additionally, we focused only on specific brain regions in volumetry analysis. A notable strength of our data set is the homogeneity of the study groups, provided by strict CBAS criteria including AD biomarkers. In order to quantify the predictive power of EPR for the identification of individuals at risk of AD, larger group studies would need to be performed and machine learning approaches utilized. Other limitations could include the sample size and lack of controls for other covariates, which may have impacted the EPR and volume associations (e.g., depression).

## Conclusions

In conclusion, this study demonstrates that aMCI due to AD and AD dementia individuals have lower EPR scores as compared to cognitively healthy participants. Given that EPR scores correlate with MMSE scores and regional temporal brain atrophy, these data suggest that EPR may be an additional tool to stage AD and/or improve early diagnosis of AD and to guide clinical and social management of individuals with cognitive impairment.

## Data Availability

The datasets used and/or analyzed during the current study are available from the corresponding author on reasonable request.
